# Standardised alcohol screening in primary health care services targeting Aboriginal and Torres Strait Islander peoples in Australia

**DOI:** 10.1186/s13722-018-0108-2

**Published:** 2018-03-29

**Authors:** M. Mofizul Islam, Helen T. Oni, K. S. Kylie Lee, Noel Hayman, Scott Wilson, Kristie Harrison, Beth Hummerston, Rowena Ivers, Katherine M. Conigrave

**Affiliations:** 1 Department of Public Health, La Trobe University, Melbourne, Australia; 2 NHMRC Centre of Research Excellence: Indigenous Health and Alcohol, Sydney, Australia; 3 Indigenous Health and Substance Use, Discipline of Addiction Medicine, Sydney Medical School, The University of Sydney, Sydney, Australia; 4 Southern Queensland Centre of Excellence in Aboriginal and Torres Strait Islander Primary Health Care, Inala, Australia; 5 Faculty of Medicine, University of Queensland, Brisbane, Australia; 6 School of Medicine, Griffith University, Brisbane, Australia; 7 Aboriginal Drug and Alcohol Council SA, Underdale, South Australia Australia; 8 Discipline of Addiction Medicine, Sydney Medical School, The University of Sydney, Sydney, Australia; 9 Sydney School of Public Health, The University of Sydney, Sydney, Australia; 10 Alcohol Treatment Project, Aboriginal Health Council of South Australia Ltd, Adelaide, Australia; 11 Illawarra Aboriginal Medical Service, Wollongong, Australia; 12 Graduate School of Medicine, University of Wollongong, Wollongong, Australia; 13 Royal Prince Alfred Hospital, Sydney Local Health District, Camperdown, Australia

**Keywords:** Alcohol screening, AUDIT, AUDIT-C, Indigenous, IRIS, Aboriginal and/or Torres Strait Islander

## Abstract

**Introduction and aims:**

Aboriginal and Torres Strait Islander Community Controlled Health Services (ACCHSs) around Australia have been asked to standardise screening for unhealthy drinking. Accordingly, screening with the 3-item AUDIT-C (Alcohol Use Disorders Identification Test—Consumption) tool has become a national key performance indicator. Here we provide an overview of suitability of AUDIT-C and other brief alcohol screening tools for use in ACCHSs.

**Methods:**

All peer-reviewed literature providing original data on validity, acceptability or feasibility of alcohol screening tools among Indigenous Australians was reviewed. Narrative synthesis was used to identify themes and integrate results.

**Results:**

Three screening tools—full AUDIT, AUDIT-3 (third question of AUDIT) and CAGE (Cut-down, Annoyed, Guilty and Eye-opener) have been validated against other consumption measures, and found to correspond well. Short forms of AUDIT have also been found to compare well with full AUDIT, and were preferred by primary care staff. Help was often required with converting consumption into standard drinks. Researchers commented that AUDIT and its short forms prompted reflection on drinking. Another tool, the Indigenous Risk Impact Screen (IRIS), jointly screens for alcohol, drug and mental health risk, but is relatively long (13 items). IRIS has been validated against dependence scales. AUDIT, IRIS and CAGE have a greater focus on dependence than on hazardous or harmful consumption.

**Discussion and conclusions:**

Detection of unhealthy drinking before harms occur is a goal of screening, so AUDIT-C offers advantages over tools like IRIS or CAGE which focus on dependence. AUDIT-C’s brevity suits integration with general health screening. Further research is needed on facilitating implementation of systematic alcohol screening into Indigenous primary healthcare.

## Background

Although Aboriginal and Torres Strait Islander (Indigenous) Australians are more likely to abstain from drinking alcohol than other Australians, a greater proportion of those who do consume alcohol engage in risky drinking [1]. These patterns of drinking have historical roots and often reflect ongoing experience of dispossession, marginalisation, disadvantage, racism, grief, trauma and loss. As a result, Indigenous Australians are up to eight times more likely to be hospitalised and five times more likely to die from an alcohol-related condition than their non-Indigenous counterparts [1].

Screening for unhealthy alcohol use (drinking over recommended limits or alcohol use disorders) allows identification of people who are at risk of developing a health or social problem due to alcohol even if they have not experienced such a problem. Health problems linked to alcohol include common conditions such as raised blood sugar or blood pressure, poor sleep, anxiety or depression or alcohol dependence. The screening process itself can give the individual a chance to reflect on their consumption and may result in reduced consumption [2, 3]. In addition, a brief structured conversation on drinking (brief intervention) has been found to result in reductions of drinking for a broad range of unhealthy alcohol use, at least in the short term [4]. A brief discussion about drinking after a ‘positive’ screen, is a cost-effective way to help individuals in primary health care settings whose drinking poses a risk to their health or wellbeing [4]. Those with alcohol dependence can also be referred to specialised drug and alcohol services if needed.

Around the world, drinkers with an alcohol use disorder (harmful use or dependence) tend to seek help late when significant harms have already occurred. There are many barriers to Indigenous Australians accessing alcohol treatment, including lack of culturally appropriate services and resources, lack of transport or childcare, and actual or perceived racism [5, 6]. These barriers may further delay help-seeking [7, 8]. Because of this, active screening and discussion of drinking is particularly important.

Aboriginal and Torres Strait Islander Community Controlled Health Services (ACCHSs) provide access to culturally appropriate and accessible services. However, in these busy primary health care services, clients often present with complex health and social needs [9]. So, it can be difficult to find time to conduct alcohol screening alongside responding to the reason for a person’s visit. Alcohol can also be a sensitive topic, because of experience of racially-based assumptions about drinking, or because of shame about alcohol-related social problems.

Alcohol screening has been included for many years in the annual Aboriginal or Torres Strait Islander health check, and reporting on clients’ drinking status has been part of national key performance indicators for ACCHSs [10]. However, the criteria used to classify an individual as a ‘safe’ or ‘unsafe’ drinker were not defined. Different health staff could have different perceptions of what drinking patterns are safe. Recently the federal government asked ACCHSs, which receive federal funding, to standardise their alcohol screening. As a result, from June 2017 all ACCHSs were asked to report results of screening using the 3-question Alcohol Use Disorders Identification Test—Consumption (AUDIT-C) [11].

AUDIT-C asks about frequency and quantity of drinking, and the frequency of drinking six or more ‘standard’ drinks (where a standard drink is 10 g ethanol in Australia). AUDIT-C has been widely validated internationally as a tool for detecting unhealthy drinking in a primary care setting. It is one of many brief screening tools that have been used globally. AUDIT-C and other alcohol screening questionnaires vary in specificity, sensitivity, cut-off score, length and ease of use. Their performance can also vary with different population subgroups [12]. Some of these screening tools, including AUDIT, AUDIT-C, CAGE (Cut-down, Annoyed, Guilty and Eye-opener) and CRAFFT (Car, Relax, Alone, Forget, Friends, Trouble) have been used among Indigenous populations in other parts of the world [13–24]. However, only a small number of studies examine their validity and acceptability in that setting [15–17].

In this paper we examine evidence for the suitability and acceptability of AUDIT-C and of alternative validated brief alcohol screening tools for routine use in primary health care services targeting Indigenous Australians.

## Methods

**Fig. 1:**
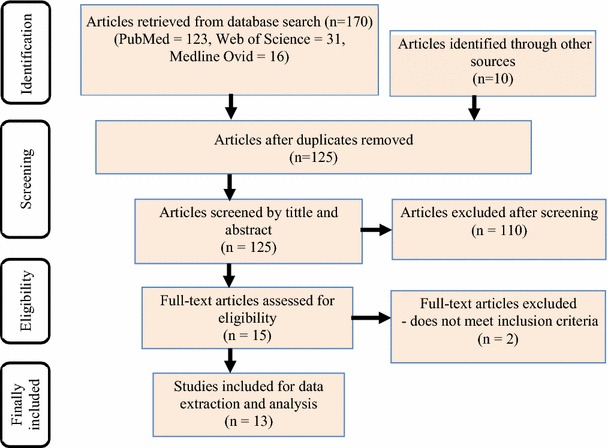
Diagram summarising procedure for selecting eligible articles for systematic review of alcohol screening among Indigenous Australians. Search terms used: Alcohol (MeSH), Aboriginal (MeSH), Australia (MeSH), Aboriginal OR Indigenous, screening, alcohol AND screening, Alcohol Use Disorders Identification Test. AUDIT-C, valid*, (((((Alcohol) AND screening) OR valid*) AND Aboriginal) AND Australia), (((((Alcohol) AND screening) OR Alcohol Use Disorders Identification Test) AND Aboriginal) AND Australia), TOPIC: (Alcohol) *AND* TOPIC: (Alcohol Use Disorders Identification Test) *AND* TOPIC: (Aboriginal) *AND* TOPIC: (Australia)

A review was conducted of all original data on validity, acceptability or feasibility of alcohol screening among Indigenous Australians published up to April 2017. A range of search terms were used in Web of Science, PubMed and MEDLINE to identify potential peer-reviewed articles (Fig. 1). Grey literature was also searched (e.g., reports, monographs and clinical guidelines) for original data on alcohol screening among Indigenous Australians using the Australian Indigenous HealthInfoNet, the Indigenous Australian Alcohol and Other Drugs Bibliographic Database and the Google Scholar search engine. Finally, hand searching of reference lists was undertaken. The literature search was conducted by the first and second author (MMI, HO), and the search approach and retrieved articles were checked by an expert librarian.

Peer-reviewed articles that provided original data on validity, acceptability or feasibility of alcohol screening tools and/or brief interventions among Aboriginal or Torres Strait Islander peoples in Australia were included. Duplicate studies were excluded. Data was extracted independently by the first author (MMI) utilising a template in line with the aims of this review. A narrative synthesis of the retrieved literature was conducted by the first (MMI) and the senior author (KC). A narrative synthesis is an approach to synthesise and summarise findings from multiple studies that relies primarily on the use of words and text; it uses a textual approach to describe the key findings extracted from the reviewed article [25, 26]. This method is suited where there is considerable diversity in the methods used in the retrieved literature, including in design and/or data collection techniques [27].

## Results

A total of 170 articles were found from searches of mainstream academic databases and an additional 10 references from other sources (Fig. 1). After applying the inclusion/exclusion criteria, 15 articles were considered and 13 were finally selected for data extraction and analysis.

The literature revealed an awareness of the need to use culturally appropriate but standardised measures for screening and assessment of alcohol use among Indigenous Australians [5, 9]. For instance, Gray et al. [9] mentions that interventions to reduce alcohol-related harm cannot simply be transferred from non-Aboriginal to Aboriginal settings. However, there were few investigations about the acceptability and validity of alcohol screening tools in ACCHSs (Table 1). A summary of the literature, which includes data on the validity, acceptability or feasibility of AUDIT and its short forms (e.g. AUDIT-C, AUDIT-3), and on CAGE, SMAST (Short Michigan Alcoholism Screening Test), IRIS and KAT (Khavari Alcohol Test) questionnaires is presented below.

**Table 1:** Research on alcohol screening tools among Indigenous Australians

References	Setting (remoteness)	Participants	Screening tool (number of items)	Study type	Some relevant observations
Skowron and Smith [36]	Port Hedland area (Remote WA)^a^	Homeless Aboriginal people (n = 162)	CAGE^b^ (4 items) SMAST^c^ (12 items)	Quantitative: CAGE compared with clinical interview on drinking	CAGE had reasonable validity in comparison with clinical interview Those with higher CAGE score drank more on previous day, and more frequently SMAST was found hard to understand by participants in a pilot study
Hunter et al. [24]	Kimberley region (Very remote, WA)	Aboriginal community members (n = 516)	CAGE (4 items) (reworded for local use of English)	Quantitative: compared with clinician interview	CAGE scores were associated with frequency of alcohol consumption Over half of ex-drinkers had a CAGE score of 2 or more
Brady et al. [29]	ACCHS (Urban)	Health staff (n = 14), clients (20)	AUDIT^d^ (10 items); Two questions: average days per week the patient drank, and amount and type of drinks per day	Mixed methods	AUDIT was reported by health workers to be long and intrusive Problems with question comprehension, when using standard English phrasing Preferred two questions on frequency and quantity
Kowalyszyn and Kelly [28]	Community setting (Remote far north Queensland)	Aboriginal community members (n = 99)	AUDIT (10 items) KAT^e^ (12 items)	Quantitative: two screening tools compared	High correlation between AUDIT and KAT responses AUDIT seemed easier for participants to complete and had more face validity Sharing of alcohol led to challenges in quantification of drinking
Schlesinger et al. [37]	Clinical and non-clinical services (Urban, regional and remote Queensland)	Aboriginal and Torres Strait Islander clients of services (n = 175)	IRIS^f^ (13 items)	Quantitative: IRIS compared with AUDIT, two dependence scales (SDS^g^, LDQ^h^), mental health scales, and interview on consumption by Aboriginal community worker	IRIS was validated as a screen for alcohol and drug, and mental health risk IRIS had good convergent validity with other scales for alcohol risk, substance use, dependence, and mental health, including AUDIT Good sensitivity for detecting men drinking 11+ drinks per occasion; some false negatives for women with 7+ drinks
Clifford and Shakeshaft [20]	ACCHSs: (One urban and one rural, in NSW)	Health staff (n = 32) and patients (n = 24)	AUDIT (10 items)	Mixed methods: survey and group interviews of staff and (separately) of patients	Staff reported: Staff and patients preferred the first three items of AUDIT (AUDIT-C) over the full AUDIT; Patients screened with AUDIT-C reportedly showed more interest in their drinking risk than those previously screened with a single question on average consumption; and Staff and patients preferred screening to be part of a routine health check rather than opportunistic
Clifford et al. [38]	Five ACCHSs (Urban and regional NSW^i^)	Health staff (n = 37)	A range of (typically unvalidated) questions that were being used by health services	Qualitative; (semi-structured group staff interviews)	Except in adult health check, screening was generally selective A range of questions were being used, and often did not quantify consumption clearly
Conigrave et al. [5]	Aboriginal community-based groups (Urban NSW)	Aboriginal group participants (n = 47)	AUDIT (10 items) Rephrased for local English use	Quantitative plus researcher observation	AUDIT seemed easy to understand, as long as help was available for individuals not comfortable with reading While AUDIT-C may be enough to identify risk, the whole AUDIT provided a chance for the drinker to reflect on impacts of their drinking Community members appeared interested to receive their AUDIT score
Lee et al. [30]	Alcohol and drug treatment service –mainstream (Urban, NSW)	Staff and Aboriginal patients (n = 21, n = 24 respectively)	AUDIT-C—modified^j^ (3 items)	Mixed methods	An interviewer asked the AUDIT-C questions in a conversational style, adapting phrasing as needed Interviewer helped quantify drinking, as challenges with numeracy, a culture of sharing alcohol, and a common interpretation of the term ‘to drink’ as ‘to drink very heavily’
Ober et al. [23]	Prisons in Queensland	Aboriginal or Torres Strait Islander inmates (n = 395)	IRIS—modified (13 items); Asking about substance use in 12-months *before* prison	Quantitative: compared against CIDI	IRIS was compared against ICD10 criteria for substance use disorders (using CIDI) IRIS had a high sensitivity (94%) and low specificity (33%)
Calabria et al. [32]	Primary care and community-based settings; (urban and regional NSW)	Aboriginal patients and community members (n = 136)	AUDIT-C (3 items) AUDIT-3 (1 item) AUDIT (10 items); Used plain English, no conversion to standard drinks	Quantitative: short forms of AUDIT compared against full AUDIT	AUDIT-C and AUDIT-3 (at appropriate cut-offs) compare favourably to full AUDIT
Gray et al. [9]	Several sites- ACCHS and community-based (urban through to remote)	Researchers of five studies; plus overview of their results	AUDIT (10 items)	Review of five alcohol studies with Aboriginal people; workshops with the researchers	Few people (Aboriginal or otherwise) have a clear understanding of a ‘standard drink’ and the amounts poured or consumed as ‘a drink’ generally Understanding cultural context is key
Noble et al. [35]	An ACCHS (Regional NSW)	Service clients (n = 188; of whom 72% were Aboriginal)	AUDIT-3m^k^; (2 items) Via touch-screen laptop	Quantitative: compared with 7-day retrospective drinking diary	81% of Aboriginal current drinkers (n = 69) were equivalently classified by the two measures (weighted kappa = 0.77, 95% CI 0.73, 0.83) 7-day diary missed 31% of current drinkers who did not consume alcohol in the past week

^a^WA: the state of Western Australia

^b^CAGE: four questions which relate to being unable to Cut down on drinking; being Annoyed by people criticising drinking, Guilt about drinking, Eye opener or morning drink to get over adverse effects

^c^SMAST: 12 items, short Michigan Alcoholism Screening Test

^d^Alcohol Use Disorders Identification Test (AUDIT):10 items, with three on consumption, three on dependence and four on harms

^e^Khavari alcohol test (KAT): 12 questions which enquire into consumption of different types of alcoholic beverages

^f^Indigenous Risk Impact Screen (IRIS)

^g^Severity of Dependence Scale (SDS)

^h^Leeds Dependence Questionnaire (LDQ)

^i^NSW: the state of New South Wales

^j^AUDIT C changes: Q2 was asked before Q1; Q3 was adapted to fit Australian drinking guideline to reduce risk from a single occasion of drinking: “How often would you have four or more standard drinks each time you drink?” Note that respondents were not asked to convert to standard drinks

^k^AUDIT 3m: The third question of AUDIT modified to fit two Australian drinking guidelines; “How often do you have MORE THAN 4 standard drinks on one occasion?” and “How often do you have MORE THAN 2 standard drinks in one day?” (visual images representing that amount are provided)

### The Alcohol Use Disorders Identification Test (AUDIT) and its short-forms

AUDIT is a 10-item screening tool that was developed and internationally validated under the auspices of the World Health Organization. It has three questions which ask about consumption (also known as AUDIT-C), three about dependence, and four about effects of drinking. AUDIT and its short-forms predominate in the sparse literature available on alcohol screening in ACCHSs.

AUDIT has been found to have good internal consistency (alpha coefficient of 0.94) and good correlation (r = 0.69) with a 12-item measure of alcohol consumption, (KAT) in remote northern Queensland [28]. However, challenges in quantifying alcohol consumption were noted, particularly given the common practice of sharing alcohol. In a New South Wales (NSW) urban setting, AUDIT was found to be acceptable and was observed to prompt reflection and provide a springboard for a conversation on drinking [5].

Despite AUDIT’s acceptability in a community setting, several mixed methods, qualitative and quantitative studies reported barriers to using AUDIT in ACCHSs. In a study in an urban ACCHS, Aboriginal health workers said that they found the full AUDIT long. Some clients were reported to be displeased when presenting to the ACCHS for one health concern and then being asked 10 seemingly unrelated questions about alcohol [29]. Staff in that ACCHS and another service expressed a strong preference for only 2–3 consumption questions instead of the full AUDIT [20, 29] (see below).

In the urban study above, Aboriginal health workers also found questions in the full AUDIT were “intrusive”, “getting too close”, and “prying into their [clients’] private life” [29]. They said that: “You need someone out[side] of the extended family [to do this screening], someone out of it all” [29]. After switching to screening for consumption only, and after 12-months implementation, staff reported screening for alcohol consumption was getting easier.

Several studies pointed out the difficulty of quantifying consumption, in particular, the difficulty of asking individuals to convert their drinking to ‘standard drinks’ when using AUDIT with its original wording [30]. Several approaches were used to help with this. Visual aids, either printed or on a computer, to show the clinician or client what the equivalent measure of a standard drink is [3, 29–31]. Three studies in urban and regional NSW used a modified version of AUDIT, which allowed respondents to record their consumption as ‘drinks’ rather than as ‘standard drinks’ [5, 30, 32]. The authors acknowledged that this approach may not be perfect, but that having a tool that was understandable and easy to administer outweighed any potential loss in accuracy. The authors were not able to examine the impact of this modification on sensitivity. In another study in an ACCHS in regional NSW, a touchscreen computer showed an image of a drinking threshold (e.g. four standard drinks was shown as an image of 1.5 × 750 ml bottles of beer) when asking a modified version of AUDIT-Q3 (frequency of drinking 2+ or 4+ drinks per day) [31]. The computer was found to be an acceptable way to conduct screening in the clinic waiting room. Another challenge with quantifying drinking, is that sharing is a cultural norm, and drinkers may sometimes report on the consumption of the entire group, rather than on their own consumption [5, 28, 33].

Some researchers reported that AUDIT Question 4 (“How often during the last year have you found that you were not able to stop drinking once you had started?”) can cause confusion, as some individuals regularly stop drinking when they run out of alcohol or money [34]. So, continuation of drinking is more reliant on supply than on presence of alcohol dependence.

The phrasing of several questions of AUDIT was adapted to local English in consultation with local Aboriginal people. For example, the local English translation of Question 7 (on guilt or regret about drinking) was different in a remote and in an urban Australian location [24, 30].

Shorter forms of AUDIT have been found acceptable in several ACCHSs. In some NSW ACCHSs the preferred short screen was AUDIT-C (the first three questions of AUDIT) [20]. In one urban ACCHS the preferred screen was a variant of only AUDIT Questions 1 and 2 (i.e. asking about number of days drinking in a week, and quantity and type of drinking) [29]. In another regional NSW study, a modification of AUDIT-3 alone was used and found to be acceptable [35].

In urban and regional NSW, recommended cut-off scores for AUDIT-C and AUDIT-3 were determined in comparison with the full AUDIT [32]. The cut-off scores selected were: for at-risk drinkers, AUDIT-C ≥ 5, AUDIT-3 ≥ 1; for high-risk drinkers, AUDIT-C ≥ 6, AUDIT-3 ≥ 2; and for likely dependent drinkers, AUDIT-C ≥ 9, AUDIT-3 ≥ 3. Adequate sensitivity and specificity were achieved for these cut-off scores for both AUDIT-C and AUDIT-3, relative to the 10-item AUDIT. The authors concluded that AUDIT-C provided nearly as good an estimate of alcohol misuse as the full AUDIT. However, no external criteria (e.g. clinical assessment) were available to assess performance of the full AUDIT.

In regional NSW, the modified version of AUDIT-3 (AUDIT-3m; Table 1) agreed well with a 1-week retrospective drinking diary [35]. However, the AUDIT-3m identified more current drinkers than the diary. The authors comment that this was because a 1-week diary did not adequately capture episodic drinking patterns.

### Other tools

The 4-item CAGE has been used among Indigenous Australians in Western Australia, sometimes with modified wording [24, 36]. CAGE was found to have reasonable validity in a remote setting, where individuals with a high score were found to have consumed significantly more alcohol on the day before interview [36]. Similarly, in a later study in very remote Western Australia, CAGE scores were associated with frequency of drinking [24]. However, in the latter study it was noted that over half of ex-drinkers scored two or more on the CAGE items [24]. In a pilot study for the above work in remote Western Australia, the SMAST was administered to 12 Aboriginal participants, but was not used further as participants had difficulty understanding its 12 questions [36].

As noted above, in a remote Queensland Aboriginal community the KAT (a 12-item scale to assess consumption) was compared with AUDIT. There was good correlation between the two measures, however AUDIT was found easier to administer and had greater face validity [28].

### The Indigenous Risk Impact Screen (IRIS)

IRIS is a 13-item tool which screens jointly for risk of alcohol use, other drug use, and mental health [37]. It was developed by Indigenous and non-Indigenous investigators. The IRIS has been reported to be acceptable and culturally appropriate and found valid in relation to recognised international questionnaires for assessing substance use dependence and mental health at the time of its development. IRIS asks about alcohol and other drugs simultaneously (e.g. “In the last 6 months have you needed to drink or use more drugs to get the effects you want?”). Its seven substance use questions focus only on aspects of dependence. There is no question on amount or frequency of consumption. In men it had high sensitivity for detecting 11+ standard drinks per occasion, but in women it had imperfect sensitivity for detecting 7+ drinks. In a subsequent study of Indigenous prison inmates in Queensland [23], a version of IRIS modified to ask about the pre-prison period was found to have high sensitivity (94%), but low specificity (33%) in detecting substance use disorders. The final six questions of IRIS screen for mental health risk and past psychological trauma. IRIS is said to be used and found to be acceptable by a range of services for Indigenous Australians [23] however it is not clear if this is primary care sections of the services, or other (e.g., mental health and wellbeing) sections.

## Discussion

Screening and early discussion of drinking is important in improving health, given the role of alcohol as a risk factor for a wide range of common conditions, such as diabetes, hypertension, cardiac arrhythmias and cancers [39, 40]. However, only a small number of screening tools have been validated for use with Indigenous Australian peoples. AUDIT and its short forms, IRIS and CAGE were all found to have validity compared to other screening tools or questions on alcohol consumption. Responses to the 12-item KAT correlated with those of AUDIT, but KAT was found less easy to use in Indigenous settings. AUDIT and its short forms were the only instruments for which data was available on feasibility of routine implementation in ACCHS primary care. Services found the full 10-item AUDIT too lengthy for busy primary care settings, and strongly preferred only 2–3 of AUDIT’s consumption questions.

### Acceptability and feasibility for screening in an ACCHS setting

ACCHSs offer a unique opportunity for screening, given their accessibility and appropriateness for Indigenous Australian peoples. However, services are dealing with many other complex health and social needs. A screening tool for use in ACCHSs needs to be acceptable, easily understood by the clients and staff, and quick to use and score [29, 38]. Anecdotally many ACCHSs have adopted AUDIT or (more often) its shorter versions and found it useful, even in remote settings. Others, particularly in remote regions, have reported challenges with quantifying consumption, which may be of a ‘stop-start’ rather than a regular pattern. Meanwhile, other services have chosen IRIS as their preferred screening tool. However, there is no publicly available data on the extent of use of either IRIS or AUDIT-C in ACCHSs, and on whether these are being used more in primary care sections of the service, or by drug and alcohol, mental health or social and emotional wellbeing units.

AUDIT-C’s brevity (at 3 items) is a major strength for the primary care context [34]. There are several reports on use of AUDIT’s short forms (1–3 items) in ACCHSs [29, 32, 35, 38]. These brief screening tools can more readily be embedded into a general clinical interview or routine health check than a 10–13 item instrument, such as the full AUDIT or IRIS. Because of AUDIT-C or AUDIT-3’s focus on consumption, these tools have good potential to detect drinking that is over recommended limits, and not necessarily causing current harms or symptoms of dependence.

Another advantage of AUDIT-C (or AUDIT) over other alcohol screening tools is that these start with a mild question (“How often do you have a drink containing alcohol?”). The response options include “never”. Given that the majority of Indigenous Australians are likely to be current non-drinkers [1], this may be more acceptable than an initial question that focuses on heavy drinking or dependence [41], which is the case with CAGE or IRIS. Only one study examined AUDIT-3 (in modified form) as a single question, and in this study, electronic delivery mode was used to visually demonstrate the drinking thresholds (e.g. How often did you drink more than this?).

IRIS was developed in clinical and non-clinical settings by and for Indigenous Australian peoples [37]. IRIS’s approach to integrated screening for alcohol, other drug use disorders and mental health risk is compatible with the holistic view of health among Indigenous Australians. Its final item: “Do past events still affect your wellbeing today?” recognises the frequency of trauma, including that inflicted by government child removal policies. Also, ending on a question about past psychological trauma may require de-briefing. In addition, all IRIS’s substance use questions focus on dependence. This means that like CAGE, it is less well suited to detecting drinking which may be above recommended limits (and so pose a risk for health), but is not currently resulting in health problems, or dependence. There is not published data available on the routine implementation of IRIS as a tool for universal screening in primary health care, but with 13 items, its length may pose challenges.

#### National and international comparability

AUDIT-C has been used in many other countries, cultures, and racial and ethnic groups [18, 19] such as African-American and Hispanic patients [42], Maori peoples [21, 22], and First Nations Canadians [43]. Because of this, AUDIT and its short forms allow comparability of screening results with other services, and with international research.

### Reported challenges of screening

Quantification of drinking was reported to be challenging in several studies [5]. This challenge affects any screening tool, such as AUDIT or its short forms which record consumption. People in ‘dry’ regions (where alcohol is prohibited) may have only episodic access to alcohol. Also, in any setting, relatively few people (Indigenous or other) have a clear understanding of the size of a ‘standard drink’, and individuals may not know the volume of a drink that they pour themselves. Non-standard containers may be used, for example wine poured into empty soft drink bottles [33]. Furthermore, sharing of drinks, educational disadvantage [44], or differing traditional approaches to numbering can add to the challenge of quantifying the amount of alcohol consumed in terms of standard drinks [30]. Hand-held iPad or interactive touch-screen computers have been used to assist participants to estimate consumption [31, 45]. These devices may also potentially reduce the time required to assess consumption [33].

Several authors pointed to challenges in understanding questionnaires if they were not translated into local use of English or local language in consultation with local Aboriginal or Torres Strait Islander people [24, 30]. Formal translation and back translation may be indicated if significant changes are required [46]. If re-wording goes beyond simple ‘translation’, then the new scale may need cross-validation or checking against external criteria [47]. Even with translation, some questions may function differently in different settings. For example, Question 7 of AUDIT asks if a person feels guilty about their drinking, but the response may reflect local community attitudes to drinking (acceptance of drinking) as much as individual regret [34].

### Areas for further research

#### The AUDIT-C cut-off score and false positives

Given the overall high prevalence of risky drinking among those who currently drink any alcohol among Indigenous Australians [48], and the challenges in accurately reporting drink size, a relatively low cut-off score (AUDIT-C ≥ 3 for women and ≥ 4 for men) is suggested. This is to ensure good sensitivity for detecting unhealthy alcohol use. These scores are lower than the nationally recommended cut-off scores for screening in the current Australian alcohol treatment guidelines (≥ 5 for both women and for men). No screening test is perfect, and with these cut-offs some clients with low risk drinking can potentially screen as a ‘false’ positive. However the recommended ‘treatment’ response for a positive result is further assessment or empathic discussion of drinking [49]. This can include clarification of recommended limits [50]. So it could be argued that such discussion may contribute to prevention and greater community-wide health literacy, regardless of the individual’s current level of risk. However, further research could assess the overall impact of false positive assessment on staff workload and attitudes to screening. Also, training and evaluation of this is needed to ensure that discussions are conducted sensitively.

Clinical assessment after a positive screen result typically involves checking the drinking history, including drink sizes. Where drinking is above recommended limits, questions can be asked about harms from drinking or evidence of dependence, such as ‘grog shakes’ or loss of control over drinking [51, 52]. Some clinicians with limited experience working with alcohol may prefer to use the remaining seven AUDIT or IRIS questions as a second stage screen for alcohol use disorders.

#### Refining the gold standard

Alcohol screening tools have typically been validated against internationally published screening or assessment instruments. However it is not clear how valid those ‘gold standards’ themselves are in an Indigenous context [33, 34, 37]. Further research is needed to refine or develop reference standards. As AUDIT-C is now recommended for routine implementation in ACCHS, it is timely to assess this tool against an acceptable and appropriate gold standard in an Indigenous context.

#### Research or evaluation of implementation

Any screening or assessment approach could benefit from pilot testing across a range of settings [33, 34], as Indigenous Australians comprise many diverse peoples, including those living with more traditional lifestyles and speaking languages other than English.

### Likely challenges in implementation and need for training

Clinicians need to be trained on how to estimate alcohol consumption, including standard drink quantities, drink sizes and sharing. There may also be cultural barriers to Indigenous health professionals asking about alcohol use when the client may be a close friend, or family or community member [29]. Approaches such as embedding the alcohol questions into a general health check, and explaining that all clients are asked them is likely to reduce sensitivity [9, 44]. Also, universal rather than targeted screening, should reduce the sensitivity over time [29].

Clinicians are likely to benefit with the provision of an aid for converting drinking into standard drink sizes. A touchscreen computer or computer ‘app’ may eventually help overcome difficulties in assessing consumption, and may also increase privacy and lessen social desirability bias [31, 33, 45, 53].

### Limitations

There is a limited evidence base of literature on alcohol screening that is specific to Indigenous Australians. Much of the screening research in Indigenous settings has included AUDIT or its short forms, so more data were available on this than on other tools. Moreover, while the findings favoured the short forms of AUDIT over other tools, estimating standard drinks in order to calculate an AUDIT-C score accurately is cumbersome in an Indigenous context. Furthermore, the synthesis of evidence in this report relied on the authors’ clinical and public health experience, so subjective judgements were needed. Thus, findings should be interpreted with caution. This review examines only validity and acceptability of brief alcohol screening tools. There remains minimal published evidence on the effectiveness of subsequent brief intervention, treatment or referral for unhealthy alcohol use in an Indigenous Australian setting [54–56]. This is an important area for further research.

## Conclusion

Research on appropriate alcohol screening tools for Indigenous Australians is sparse. However the short forms of AUDIT, including AUDIT-C appear to be suitable and valid for ACCHS primary care settings when delivered in locally appropriate language. Training may be needed to facilitate implementation, including accurate screening of consumption level, responses to a positive screening result. Embedding the screening questions into practice software will also support implementation of screening. Clients (and clinicians) should be supported to quantify drinking by an interpreter, and/or by use of visual aids and/or computer technology. Positive screening should be followed either by clinical assessment or a second stage screen (e.g. IRIS or the remaining AUDIT questions). IRIS may be valuable as an additional tool in drug and alcohol, or social and emotional wellbeing sections of ACCHSs where there may be less time pressure, and to put alcohol use in its broader context of other substance use and mental health. Given the high prevalence of alcohol-related harms, routine and regular screening in ACCHSs needs to proceed, even while consultation, research and evaluation continues to optimise screening approaches.
